# GERIATRIC SCHOLARS: INNOVATIVE SUPPORT FOR CAREGIVERS OF VETERANS WITH DEMENTIA

**DOI:** 10.1093/geroni/igae098.0844

**Published:** 2024-12-31

**Authors:** Nicholas Koufacos, Judith Howe

**Affiliations:** James J Peters VA Medical Center, Bronx, New York, United States; Ichan School of Medicine at Mount Sinai, Yonkers, New York, United States

## Abstract

As the general population ages, the number of Veterans living with dementia continues to grow. Veterans often rely on informal caregivers to manage their daily needs and ensure their health and safety. Caregivers may experience high levels of chronic stress, overwhelm, and fatigue as a result of their caregiving. Chronic stress may negatively affect the mental, physical, and emotional health of the caregiver, and consequently, the care recipient may also experience poor health outcomes. As such, it may be vitally important to promote self-care and stress-reduction among caregivers. Programs designed to support caregivers such as respite care may be difficult to access due to nationwide staffing shortages, and this is especially true in rural areas. In order to test whether an intervention designed specifically to assist caregivers with reducing stress would be effective, in June 2020, a Geriatric Scholars Program called the Rural Interdisciplinary Team Training (RITT) created an innovative expansion to their team trainings in rural areas. A palliative care certified social worker was appointed by RITT to function as the Dementia Care Coordinator (DCC) and offer virtual supportive counseling sessions and a virtual monthly support group for caregivers of Veterans with dementia. The DCC utilized the VHA’s Whole Health Approach which emphasizes mindful awareness of various domains of self-care. Responses from a survey completed by 24 participating caregivers suggested that caregivers appreciated the focus on stress-reduction and self-care, valued both the individual and group support, and that participation led to reduction in stress among participants.

